# Genome Sequence of JangDynasty, a Newly Isolated Mycobacteriophage

**DOI:** 10.1128/genomeA.00320-18

**Published:** 2018-05-24

**Authors:** Casey Jang, Nancy Kalaj, Brian Hwang, Lorelei Hughes, Connie Yang, Thomas Pak, John Kim, Dong Yoon Han, Jason Tedjakusnadi, Nicholas Fernandez, Natasha Dean, Arun Muthiah, Nathaniel B. Sutter, Arturo Diaz

**Affiliations:** a Biology Department, La Sierra University, Riverside, California, USA

## Abstract

JangDynasty is a bacteriophage that infects Mycobacterium smegmatis mc^2^155. It has a genome length of 70,883 bp, with 124 predicted open reading frames (ORFs), 42 of which have known functions. JangDynasty belongs to cluster O, and like other cluster O phages, it is a siphovirus with a prolate capsid.

## GENOME ANNOUNCEMENT

As a part of the Science Education Alliance Phage Hunters Advancing Genomics and Evolutionary Science (SEA-PHAGES) (1) program at La Sierra University (Riverside, CA), students isolated the mycobacteriophage JangDynasty from soil collected in Riverside, CA, by directly plating an environmental sample onto a lawn of Mycobacterium smegmatis mc^2^155 bacteria. JangDynasty produced clear plaques with turbid halos, while electron microscopy showed that JangDynasty has a flexible noncontractile tail and a prolate head.

Illumina sequencing revealed that the phage belongs to cluster O, has a 70,883-bp long genome, and contains a short 4-nucleotide (GTGT) 3′ single-stranded terminal extension. The G+C content of the JangDynasty genome was 65.4%, similar to that of its host, M. smegmatis. Single-end run reads of 140 bp were assembled using Newbler to give a contig with 1,772-fold coverage. A total of 124 ORFs, 66 of which are leftwards transcribed, were identified by autoannotation using Glimmer (2) and GeneMark (3), followed by manual inspection and annotation revision. HHPred, BLASTp, and Phamerator (4) were used to assign functions to 42 of the 124 predicted genes.

The mycobacteriophage JangDynasty is closely related to the phages Familton, Catdawg, Corndog, Dylan, YungJamal, and Firecracker, and it joins them in cluster O (5). An NCBI nucleotide BLAST search indicates that JangDynasty’s closest relative is Familton. Although the genomes of JangDynasty and Familton are 99% identical, there are partial nonalignments due to the JangDynasty genome being 924 bp shorter than that of Familton. In part due to these gaps, genes in JangDynasty and Familton have different arrangements, indicating gene mosaicism between genomes within the same cluster.

As is the case for other cluster O phages, the genome of JangDynasty contains three blocks of genes. The first is a group of 10 leftwards-transcribed genes that includes two cytosine methylase genes and an endonuclease gene. The second is a group of rightwards-transcribed genes (genes 11 to 72) that contains the virion structure and assembly genes, as well as lysin and holin genes. Interestingly, genes encoding *O*-methyltransferase (gene 33), two glycosyltransferases (genes 34 and 35), and *N*-acetylglucosaminyltransferase (gene 36) were found between the portal and capsid maturation protease genes. Moreover, within the second group of mostly rightwards-transcribed genes there are two blocks of leftwards-transcribed genes. The third block of genes (genes 73 to 123) is transcribed leftwards and includes genes encoding the DNA polymerase III sliding clamp, an AAA ATPase, a Ku-like protein, and a ParB-like domain protein. Finally, a single gene at the extreme right of the genome is transcribed rightwards.

### Accession number(s).

The complete genome sequence of JangDynasty is available from GenBank under the accession number MG872838.
